# Impact of Inclusion of Multicomponent Synbiotic Russian Holstein Dairy Cow's Rations on Milk Yield, Rumen Fermentation, and Some Blood Biochemical Parameters

**DOI:** 10.3389/fvets.2022.884177

**Published:** 2022-07-14

**Authors:** Vladimir I. Trukhachev, Nikolai P. Buryakov, Sergey O. Shapovalov, Aleksandr N. Shvydkov, Maria A. Buryakova, Irina V. Khardik, Mohamed M. Fathala, Oksana E. Komarova, Dmitrii E. Aleshin

**Affiliations:** ^1^Department of Feeding Animals, Institute of Animal Science and Biology, Russian State Agrarian University - Moscow Timiryazev Agricultural Academy, Moscow, Russia; ^2^Department of Breeding, Feeding and Private Animal Science, Novosibirsk State Agrarian University, Novosibirsk, Russia; ^3^Faculty of Veterinary Medicine, Animal Husbandry and Wealth Development Department, Alexandria University, Alexandria, Egypt

**Keywords:** Russian Holstein, multicomponent symbiotic, milk yield, rumen fermentation, hematological and biochemical indicators

## Abstract

The purpose of this study was to appraise the effect of the inclusion of multicomponent synbiotic “Kormomix® Rumin” in feeding lactating Holstein cows on milk productivity, indicators of rumen fermentation metabolism, and some hematological and biochemical parameters of the blood. For this study, 40 highly productive Russian Holstein cows were selected according to their productivity, physiological condition, live weight, and age. They were divided into four groups (10 heads/each). All animals received the basal total mixed ration (TMR), which was balanced and corresponded to the nutritional requirements for cows during the milking period with a milk yield of 36 kg/daily. The first group (control) fed basal (TMR) only while the 2nd, 3rd, and 4th group fed the basal (TMR) supplemented with a multicomponent synbiotic “Kormomix® Rumin” in amounts 25, 50, and 75 g/head/day, respectively, which was administered manually and individually after morning feeding daily and mixing carefully together with the concentrates directly after calving until 120 DIM. Milk, ruminal fluid, and blood samples were collected for studying the studied parameters. The highest values in all studied milk parameters were recorded in the 2nd experimental group but the differences were not significant. The inclusion of “Kormomix® Rumin” increased significantly the synthesis of volatile fatty acids in the 2nd experimental group (9.38 vs. 7.04 mmol/100 ml) in the control group. The level of serum α-Amylase (total) decreased significantly in the 2nd experimental group compared with other groups. The urea level recorded the lowest value in the control group, while the urea/creatinine ratio recorded the lowest value in the 4th group and the differences were significant when compared with the 2nd group. Accordingly, the inclusion of synbiotic “Kormomix® Rumin” in the diets of lactating cows has no impact on milk production. Whereas, it improves the intensity of rumen fermentation, which contributes to more efficient utilization of feed without any harmful effects on blood traits. Moreover, the recommended dose for use in their diets is 25 g/head/day.

## Introduction

The process of developing dairy cattle breeding in Russia has undergone significant changes in recent years. The milk productivity of cows has increased up to 8,000–10,000 kg of milk/cow/year. However, such realization of genetic potential is possible only with a systematic approach providing a unique attitude toward optimization of feeding conditions and considering the biological peculiarities and metabolism of highly productive animals in different physiological periods (1–4).

One of the important causes of culling highly productive cows is the negative effects resulting from metabolic disorders. These metabolic-related diseases in dairy cattle appear during the period of intensive milk synthesis (mainly in the 1st lactation phase). The linking between the rumen microflora and the animal's body is relatively constant, but its equilibrium depends on one hand on the physiological and immunological status of the body, and on the other hand, on the quantitative and species composition as well as the biochemical activity of rumen microorganisms (5–8).

When changing the feeding regime or the ration composition, upon changing from drying to milking, it takes several days for the organisms to adapt to the change in the proportions of volatile fatty acids in the rumen. During this period, the activity of cellulolytic bacteria and fungi may be inhibited. In this case, the digestibility of structural and nonstructural carbohydrates is reduced, as well as, protein and lipids, which will eventually be reflected in the reduction of feeding efficiency of cows with genetically laid high productivity (5, 9). Also, the use of a limited set of feeds, violations in the technology of feed preparation, improper grinding of feed products, poor weather conditions, animal stress during rearing, and housing technology, all can lead to inaccessibility or poor assimilation of nutrients in the diet (10, 11).

The use of feed additives with antibiotics as growth promotors in feeding high-yielding animals has been forbidden by the European Union since 2006. The use of various chemical and antibiotic-like feed additives in the diets of high-yielding Holstein cows to obtain ecologically safe food animal products in feeding high-yielding animals is undesirable. In this regard, finding alternative feed additives to prevent health problems, to increase productivity and economic efficiency is necessary (8, 12–14).

These alternatives include digestive enzymes, probiotics, prebiotics, phytobiotics, plant extracts, and multicomponent feed additives with synbiotic action. Alternative feed additives possessing biologically active substances and contributing to better utilization of the nutrients in the ration and obtaining ecologically safe milk are multicomponent synbiotics (feed additives with live microorganisms and enzymes). Synbiotic is defined as “a mixture of probiotics and prebiotics that beneficially affects the host by improving the survival and implantation of live microbial dietary supplements in the gastrointestinal tract, by selectively stimulating the growth and/or activating the metabolism of one or a limited number of health-promoting bacteria, and thus improving host welfare” (15). Multicomponent synbiotics contain both probiotics and prebiotics in addition to enzymes. Usage of synbiotics helps increase the digestibility of structural carbohydrates, cow productivity, and milk quality and optimizes the body's metabolic processes (4, 7, 13).

At present, the use of multicomponent feed additives based on probiotic cultures containing *Bacillus subtilis* and *Bacillus licheniformis*, which regulate the rumen microbial synthesis and allow reducing the adverse effects of evolving digestive system diseases of young and adult animals, as well as the consequences of feeding low-quality feed, is practiced worldwide (4, 5, 13). Moreover, the inclusion of feed additives of pro- and prebiotic action (synbiotics) contributes to increasing the productivity of cows, improving the quality of milk, and the intensity of rumen digestion (16, 17) had shown that, the use of *B. subtilis* and *B. licheniformis*, complex enzymes, and probiotic cultures positively affects animal productivity and stimulates the fermentation of nutrients in the rumen of highly productive dairy cows.

Therefore, the perspective directions of research are the provision of multicomponent feed additive contains (probiotic, prebiotic, and enzymes) to use nutrients and energy from different components of the ration more effectively on the bases of natural physiological processes in the rumen by supporting and stimulating fermentation processes in the rumen (18). Consequently, our research aimed to appraise the influence of inclusion of multicomponent synbiotic “Kormomix® Rumin” as a feed additive in the ration of the lactating Holstein cows on the milk productivity, indicators of rumen fermentation metabolism as rumen pH, the concentration of volatile fatty acids (VFA) and ammonia, as well as, some hematological and biochemical blood parameters.

## Materials and Methods

### Characteristics of Objects and Conditions of Research

The research was conducted from December 2020 to June 2021 on a farm belonging to the joint-stock company “Plemkhoz Naro-Osanovsky” of the Odintsovsky district of the Moscow region, Russia. The experiment was conducted according to methods approved by the scientific council of the Institute of Zoology and Biology of the RSAU – MTAA (Protocol №. 198 from 12.10.2020). The animal study was reviewed and approved by the Bioethics Commission of the Institute of Animal Science and Biology of the Russian State Agrarian University — Moscow Timiryazev Agricultural Academy (Protocol № 2021-4 from 12.10.2021).

Forty highly productive Holstein dairy cows during the dry period (3 weeks before calving), giving into account their origin, age, live weight, physiological condition, and milk production. The animals were clinically healthy and kept under the same conditions throughout the experiment (3 weeks before calving until the end of the stage of lactation (120 DIM)). Cows were milked three times/day (3×).

All animals under the study consumed the same total mixed ration (TMR), which was nutritionally balanced and corresponded to the feeding rate for cows during the milking period with a milk yield of 36 kg of milk per day. The first group of animals served as control and received only the basic (TMR) consistent with the normal standards of feeding highly productive cows (Federal Research Center for Animal Husbandry named after Academy Member L.K. Ernst (19) (Tables 1, 2). while the 2nd, 3rd, and the 4th group fed the balanced (TMR) supplemented with the multicomponent synbiotic “Kormomix® Rumin” in amounts (25, 50, and 75 g/head/day) respectively until the end of the 1st phase of lactation. Administration of multicomponent synbiotic “Kormomix® Rumin” was done manually and individually to each animal after the morning feeding and mixed thoroughly and carefully together with the concentrate once/daily.

**Table 1:** Basal (TMR) composition fed to Russian Holstein cows.

**Ingredients**	**Amount/kg/day**
Meadow bluegrass hay (*Poa pratensis L*.)	1.0
Corn silage	24.0
Grain and legume haylage	13.0
Beer pellet (fresh)	6.0
Beet pulp (fresh)	3.0
Compound feed	10.5
Sunflower cake	1.5
Sodium chloride	0.17
Limestone (calcium carbonate)	0.10
Disodium phosphate	0.10

**Table 2:** Nutritional value of basal (TMR) fed to Russian Holstein cows/daily.

**Indication**	**Feeding rate (FRC for Animal Husbandry, 2016)**	**Nutritional value of diet/daily**
Metabolic energy (ME), MJ	251.0	254.0
Dry matter, kg	22.7	24.6
Crude protein, g	3,980.0	4,067.0
Digestible protein, g	2,885.5	3,008.2
Degradable protein, g	2,408.5	2,896.2
Undegradable protein, g	1,572.0	1,180.5
Crude fiber, g	3,714.5	5,219.0
Neutral detergent fiber (NDF), g	7,604.0	11,295.0
Starch, g	4,646.0	3,613.2
Sugar, g	2,102.0	803.4
Crude fat, g	956.0	1,210.0
Calcium, g	194.0	227.1
Phosphorus, g	140.5	137.6
Magnesium, g	44.5	56.1
Potassium, g	194.0	293.4
Sulfur, g	60.0	65.7
Iron, mg	2,245.5	5,666.3
Copper, mg	305.5	532.0
Zinc, mg	1,949.5	1,821.3
Manganese, mg	1,990.5	2,118.5
Cobalt, mg	25.5	24.7
Iodine, mg	28.3	37.6
Carotene, mg	1,399.0	779.3
Vitamin A, thousands of IU	223.0	262.5
Vitamin D, thousands of IU	28.5	29.31
Vitamin E, mg	954.0	2,105.3

**Figure 1:**
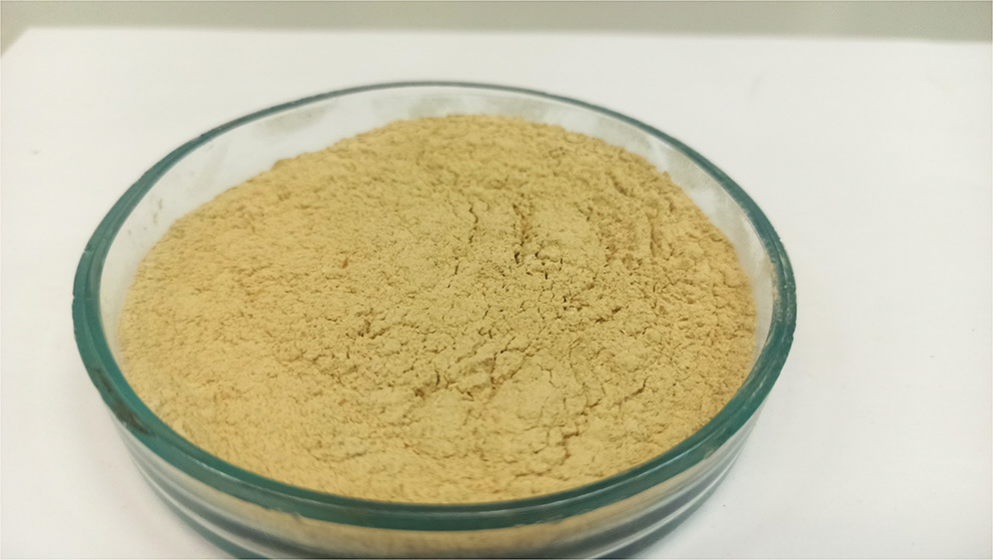
Multicomponent synbiotic “Kormomix® Rumin”.

Multicomponent synbiotic “Kormomix® Rumin” consisted of an enzyme mixture that includes amylase, cellulase, xylanase, β-gluconate, protease, phytase, live probiotic cultures (*B. subtilis* and *B. licheniformis*), prebiotic culture based on mannanoligosaccharides, and mineral filler (Figure 1) (silicon dioxide). The nutritional value of “Kormomix® Rumin” up to 100 g, is very low and will not significantly affect the composition of the diet. However, it contains many micro and macro elements. Because the action of this additive is aimed to manipulate the rumen fermentation to increase the efficiency of feed conversion and animal productivity. The Multicomponent synbiotic “Kormomix® Rumin” as a product was manufactured by LLC PO “Sibbiofarm” company (Berdsk, Russia).

Animal rations were balanced using the Feed Optima program (v. 2020.8.17251) to satisfy cows' needs (energy, protein, lipids, carbohydrates, vitamins, and minerals) during the early lactation period (120 DIM). Feed samples were sent to the Cherkizovo Research and Testing Center (Yakovlevskoe village, Troitskiy Autonomous District, Moscow) for measuring the chemical composition of the (TMR) fed to the experimental animals.

Milk samples were collected (from all dairy cows under the study) in a personal container and stored in a refrigerator at 4°C until sent to the laboratory. Fat and protein content in milk was determined in the laboratory of selective milk quality control of the regional information and selective breeding center of JSC “Moskovskoe” for breeding work (Noginsk, Moscow region) on a Combi Foss FT+ device. For the objectivity of measurements, we used reference milk samples intended for metrological control (calibration), made according to international standards: the mass fraction of fat according to ISO 1211-2012. 2446-2009; mass fraction of protein according to ISO 8968-1-2008. Control milking and milk tests were measured every 10 days. The gross yield of protein and fat in the milk of cows was calculated. Daily and gross milk yields of natural and 4% FCM were estimated based on control milkings. The gross milk yield of 4% FCM was calculated according to the formula proposed by (20) based on the gross daily milk yield of natural fat and the chemical composition of milk.

At the age of 3 months of lactation (90 DIM), blood samples were collected 3 h after the morning feeding on the subcostal vein (three cows from each group) into vacuum tubes with a coagulant coagulation activator (for obtaining blood serum for estimation of some biochemical parameters by the campaign of Zhejiang Gongdong Medical Technology Co., Ltd., China) and tubes with K3 EDTA (whole blood) for obtaining whole blood in order to estimate of hematological parameters on an ABC VET analyzer (Horiba ABZ. France) using “UniGem” reagent kits (Reamed, Russia). Biochemical blood parameters were determined at a certified independent veterinary laboratory (Moscow) on a Beckman Coulter AU 480 device (Beckman Coulter. Inc., USA).

**Figure 2:**
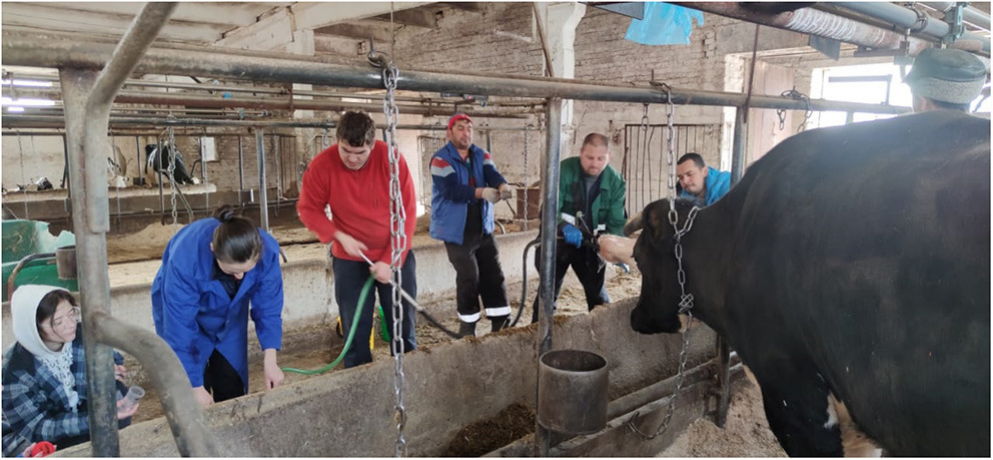
Ruminal fluid sample collection.

Samples of ruminal fluid were drawn after feeding using a transesophageal tube (rumen probe) according to the method of (21) approximately 3 h after feeding, considering that the maximum amount of saliva enters the ruminal fluid sample at the beginning of probing (Figure 2). The first portion (200 ml) was not used for analysis to determine volatile fatty acids (VFA) concentration, ammonia, and pH. Physio-chemical parameters of rumen fluid were determined in the laboratory of chemical and analytical studies of the department of physiology and biochemistry of farm animals of the Federal Research Center for Animal Husbandry named after Academy Member L.K. Ernst (Podolsk, Moscow region). In the rumen content, pH was determined directly after collection on an Aquilon 420 pH meter (Aquilon, Russia). During samples transportation from the farm to the lab, the samples were placed in a bag with refrigerating elements. The level of volatile fatty acids and ammonia was determined 2 h after sample collection. The rumen contents were then filtered through four layers of gauze and in the liquid part, the total amount of volatile fatty acids was determined by steam distillation in a Markham distillation unit apparatus and the concentration of ammonia nitrogen by the micro-diffusion method according to (22).

### Statistical Analysis

Data were statistically analyzed using the SPSS program (23), according to the following statistical model:

Yij = μ + Gi + Eij

Yij, is an observed value of the dependent variable; μ, is a constant common to all observations; Gi, is an effect due to ith treatment (**1**^**st**^ = 0 g Kormomix® Rumin), (**2**^**nd**^ = 25 g Kormomix® Rumin), (**3**^**rd**^ = 50 g Kormomix® Rumin), (**4**^**th**^ = 75 g Kormomix® Rumin); Eij, A random deviation due to unexplained sources of variation.

Percentage data were subjected to the arcsine value. Duncan's multiple range tests were used for multiple comparisons among means at (*P* < 0.05) (24). Kolmogorov–Smirnov's test was used to test the data's normal distribution.

## Results

### Indicators of Cow's Productivity and Milk Quality

Results of milk productivity indicators in the early lactation period when supplementing multicomponent synbiotic “Kormomix® Rumin” are presented in Table 3. The average daily milk yield and gross milk yield of natural fat were not significantly different (*P* > 0.05) among treatments, with the highest values at (33.87 & 4,064.80 kg) in the 2nd experimental group while the lowest values at (31.62 & 3,794.60 kg) in the control group Table 3. The same pattern was recorded in the case of the gross milk fat yield and milk protein yield.

**Table 3:** Milk productivity of cows in early lactation period (120 DIM).

**Indicators**	**Experimental groups**					
	**1st**	**2nd**	**3rd**	**4th**	**SEM**	***P*-Value**
Gross milk yield, kg/head	3,794.60	4,064.80	3,936.70	4,001.40	*46.32*	*0.198*
Daily milk yield, kg/head	31.62	33.87	32.81	33.35	*0.39*	*0.199*
Gross milk fat yield, kg/head	153.04	164.54	158.74	161.17	*2.27*	*0.343*
Gross milk protein yield, kg/head	122.53	133.50	127.95	130.39	*1.81*	*0.180*
Gross milk yield of 4% fat, kg/head	3,810.22	4,094.22	3,955.43	4,017.61	*50.04*	*0.231*
Daily milk yield of 4% fat milk, kg/head	31.75	34.12	32.96	33.48	*0.42*	*0.230*
Mass fraction of milk fat, %	4.02	4.05	4.04	4.04	*0.04*	*0.995*
Mass fraction of milk protein, %	3.23	3.29	3.25	3.26	*0.03*	*0.904*

*Values are expressed as means*.

*SEM, standard errors of the mean*.

The highest value for the average daily milk yield and gross milk yield of 4 FCM% was recorded in the 2nd experimental group and represented (34.12 & 4,094.22 kg) while the lowest values were recorded in the control group and represented (31.75 & 3,810.22 kg), respectively. Similarly, in the case of mass fraction of milk fat and protein % also, the highest values were recorded by the cows of the 2nd experimental group and represented (4.05 & 3.29 %). While the lowest were recorded by the control group and represented (4.02 & 3.23%), respectively, and the differences were not significantly differed (*P* > 0.05).

### Indicators of Fermentation in the Rumen

Results that summarize the effect of multicomponent synbiotic “Kormomix® Rumin” inclusion on rumen fermentation indicators are presented in Table 4.

**Table 4:** Indicators of rumen fermentation (metabolism).

**Indicators**	**Unit of measurement**	**Experimental groups**					
		**1st**	**2nd**	**3rd**	**4th**	**SEM**	***P*-Value**
Concentration hydrogen ions (pH)	Unit pH	7.10	6.89	6.99	6.87	*0.08*	*0.785*
Volatile fatty acids (VFA)	mmol/100 ml	7.04^b^	9.38^a^	8.40^ab^	8.86^ab^	*0.35*	*0.049*
Ammonia	Mg/dl	7.65	8.32	9.60	10.01	*0.69*	*0.654*

*Values are expressed as means*.

*SEM, standard errors of the mean*.

*Means denoted within the same row with different superscripts are significant (P < 0.05)*.

*a and b are indicating letter to the significance between means*.

The rumen fluid pH of all cows was within 6.87–7.10, which is located within the physiological norm. The total VFA content in the rumen of supplemented cows with multicomponent synbiotic “Kormomix® Rumin” exceeded the control group by 2.34, 1.36, and 1.82 mmol/100 ml, respectively. In the 2nd group, the cows were fed (25 g/head/day) multicomponent synbiotic “Kormomix® Rumin” in their diet had the highest VFA concentration, which represented (9.38 mmol/100 ml) and reliable difference (*P* < 0.05) compared to the control group (7.04 mmol/100 ml). Moreover, the level of ammonia in the ruminal content of supplemented groups was not significantly different among treatments, with the highest value at (10.01 mg/dl) in the 4th experimental group while the lowest at (7.65 mg/dl) in the control group.

### Morpho Biochemical Blood Parameters

#### Biochemical Blood Parameters

Results of biochemical blood parameters when supplementing multicomponent synbiotic “Kormomix® Rumin” are shown in Table 5. Blood glucose concentration recorded non-significant differences between different groups and ranged from 3.17 to 3.27 mmol/L.

**Table 5:** Biochemical parameters of cow's blood (90 DIM).

**Parameters**	**Unit of measurement**	**Experimental groups**					
		**1^**st**^**	**2^**nd**^**	**3^**rd**^**	**4^**th**^**	**SEM**	***P*-Value**
**Energy metabolism**							
Glucose	mmol/L	3.27	3.17	3.17	3.27	*0.06*	*0.908*
α-Amylase (total)	U/L	122.33^a^	52.67^b^	107.67^a^	87.33^ab^	*10.02*	*0.043*
Lactate dehydrogenase	U/L	944.33	1,058.00	874.67	918.33	*31.98*	*0.218*
Creatinine	mcmol/L	72.33	79.33	352.33	73.33	*69.93*	*0.450*
Creatine phosphokinase	U/L	157.00	353.00	189.33	151.00	*46.18*	*0.409*
**Protein metabolism**							
Total protein	g/L	82.80	74.10	77.80	73.27	*2.02*	*0.360*
Albumin	g/L	33.03	34.20	31.63	33.67	*1.08*	*0.887*
Globulin	g/L	49.77	39.90	46.17	39.60	*2.86*	*0.584*
Urea	mmol/L	3.73^ab^	4.20^a^	3.37^ab^	2.83^b^	*0.22*	*0.048*
Aspartate Amino transferase (AST)	U/L	84.67	90.33	88.67	75.00	*3.51*	*0.464*
Alanine Amino transferase (ALT)	U/L	28.00	30.33	30.33	29.00	*1.66*	*0.965*
De Ritis ratio		3.13	3.10	2.97	2.60	*0.13*	*0.488*
Albumin/Globulin		0.74	0.86	0.72	0.85	*0.06*	*0.812*
Ratio Urea/Creatinine		27.17^ab^	28.50^a^	22.97^ab^	20.43^b^	*1.37*	*0.049*
Gamma-glutamyl transferase	U/L	83.83	34.10	30.20	24.87	*15.45*	*0.566*

*Values are expressed as means*.

*SEM, standard errors of the mean*.

*Means denoted within the same row with different superscripts are significant (P < 0.05)*.

a and b *are indicating letter to the significance between means*.

Concerning total alpha-amylase as a criterion for energy metabolism it recorded the highest value in the blood of the control group. The differences were significant in comparison with the 2nd experimental group and represented (122.33 vs. 52.67 mmol/L). Regarding creatine phosphokinase (CPK), the differences were not significantly different and the highest value was recorded in the 2nd experimental group and represented (353.00 U/L).

When analyzing the protein metabolism data, significant differences were obtained in urea concentration and urea to creatinine ratio among cows in the 2nd and 4th experimental groups and represented (4.20 & 28.50 vs. 2.83 & 20.43), respectively.

#### Morphological Composition of the Blood of Dairy Cows

Analyzing results of morphological studies of blood at (90 DIM) revealed the superiority of Russian Holstein dairy cows supplemented with multicomponent synbiotic “Kormomix® Rumin” in their diets when compared to the control group (Table 6).

**Table 6:** Morphological composition of the blood of dairy cows.

**Indication**	**Unit of measurement**	**Experimental groups**					
		**1st**	**2nd**	**3rd**	**4th**	**SEM**	***P*-Value**
**Leukocytes**	10^9^/L	8.40	6.03	7.24	6.7	*0.93*	*0.875*
**Erythrocytes**	10^12^/L	6.30	6.70	7.13	7.28	*0.20*	*0.341*
**Hemoglobin**	g/L	81.25	85.97	80.90	81.97	*1.32*	*0.545*
**Hematocrit**	%	29.80	34.27	33.20	34.30	*0.97*	*0.344*

*Values are expressed as means*.

*SEM, standard errors of the mean*.

*Values differ non-significantly (p > 0.05)*.

The range of the number of leukocytes in the blood of experimental groups is (6.03–7.24 *vs*. 8.40^*^10^9^/L) in control. The maximum level of hemoglobin was recorded by the 2nd group in which the dairy cows were fed with (25 g/head/day) of multicomponent synbiotic “Kormomix® Rumin” in their diets and represented (85.97 g/L). Hematocrit percentage was not significantly different (*P* > 0.05) among treatments, with the highest value at 34.30% in the 4th experimental group and the lowest value at 29.80% in the control group.

## Discussion

### Indicators of Cow's Productivity and Milk Quality

The inclusion of multicomponent synbiotic “Kormomix® Rumin” in the diets of Russian Holstein cows under the study affected non significantly the fat and protein content in milk. Our results disagree with those obtained by (25, 26); they recorded an increase in the percentage and yield of milk protein in early lactating cows fed supplemental *B. subtilis*. Feeding the multicomponent synbiotic “Kormomix® Rumin” produced an additional 4% FCM in supplemented groups of Holstein dairy cows when compared with the control. Still, the difference among treatments was not significantly different. Consequently, we can assume that the inclusion of multicomponent synbiotic “Kormomix® Rumin” had no impact on milk productivity parameters compared with the control group. Our results come in the opposite direction from those obtained by (27), who reported that milk yield increased in response to supplementation of *B. subtilis*. They mentioned that cows fed 2 × 10^11^ CFU of *B. subtilis* daily produced 1.7 kg more milk than the control group. Our results also did not match those obtained by (25, 26) they reported that the milk yield increased by (3.3 and 3.4 kg/day), respectively, compared to the control group when early lactating cows' diets.

### Indicators of Fermentation in the Rumen

In the rumen content such indices as the concentration of hydrogen ions (pH), the total amount of VFA and ammonia from different sides reflect the efficiency of the fermentation process. There was no impact on ruminal pH in supplemented cows with multicomponent synbiotic “Kormomix® Rumin” which may be due to more intensive carbohydrate fermentation with the formation of VFA as well, the ruminal fluid was likely taken in the morning where there was an insufficient number of carbonates in the saliva. Thus, we assumed that the inclusion of multicomponent synbiotic “Kormomix® Rumin” did not affect ruminal pH. This result follows those obtained by (18) who found no effects on ruminal pH when bacterial probiotics were administered to dairy cows suffering subacute ruminal acidosis.

Russian Holstein dairy cows fed (25 g/head/day) multicomponent synbiotic “Kormomix® Rumin” in their diet had the highest VFA concentration which differed (*P* < 0.05) from the control group. This may be attributed to the more intense digestibility of carbohydrates in the rumen of these cows. This result comes in the same way as those obtained by (7) they recorded a significant difference in total VFA between supplemented dairy cows with the 40 g of *Saccharomyces cerevisiae* in addition to 40 g of *Lactobacillus plantarum* and the control group. Conversely, the present finding contrasts with those obtained by (28). They mentioned that supplementation of bacteria-based probiotics revealed no difference in VFA concentration among experimental groups.

With regard to the level of ammonia in the ruminal content of supplemented groups, it was not significantly different among groups when compared to the control group. This finding disagrees with those obtained by (29) which recorded that treatment with feeding *Lactobacilli* (LAB) probiotic of the diet revealed significantly higher concentrations of ruminal ammonia when compared with non-supplemented animals.

### Morphobiochemical Blood Parameters

The quantitative characteristics of biochemical blood parameters are one of the tools for controlling the nutrient and energy requirements of the body of animals. Blood glucose concentration recorded non-significant differences between different groups. Total alpha-amylase as a criterion for energy metabolism recorded the highest value in the blood of the control group and the lowest value in the 2nd experimental group. At the same time, the differences between the two groups were significant (30) mentioned that, the normal physiological limit of alpha-amylase in the blood of dairy cows (15–107 U/L). The addition of dietary digestive enzymes may improve the digestion of intestinal nutrients and provide additional nutrients for the development of normal microbiota. Intestinal microorganisms also play a vital role in the process of nutrient digestion, and can also affect the secretion of digestive enzymes (31).

Probably, the decrease in total alpha-amylase content is due to the optimal ratio of physiologically beneficial and pathogenic bacteria, the immune system, and the intestinal epithelial barrier, which in case of feeding disorders can lead to dysbiosis (disruption of the microbiota homeostasis). Due to the violation of the integrity of the intestinal mucosa, pathogenic microflora can enter the pancreas. The pancreas does not have its own microbiota, and therefore inflammatory and tumor processes affecting the gland may be associated with intestinal dysbiosis (32–34), which in turn is the cause of increases in the content of pancreatic enzymes, including amylase, in the blood. The inclusion of an additive based on probiotic and prebiotic cultures contributes to the improvement of the physico-chemical parameters of the rumen and in further work, the microbial community of the rumen of lactating cows will be studied. There is the fact that amylase in the blood when increased, it indicates a destructive effect on the intestines or pancreas. Thus, the lower level of amylase in the blood of supplemented cows represented a positive effect of the supplemental feed additive which means that the cows' bodies received additional metabolites of rumen fermentation, strengthening the metabolic and immune status of animals (35).

The observed creatinine concentrations indicate an increase in nitrogen metabolism in general, which is due to the physiological state of animals and is consistent with data on milk productivity and the chemical composition of milk. The decrease in the urea/creatinine ratio in the blood during the period is probably due to the high consumption of urea for the synthesis of microbial protein in the rumen and the use of muscle proteins for synthetic processes in the mammary gland of highly productive cows (3). The ratio of urea/creatinine intensively affects nitrogen metabolism and reflects the state of the kidneys the higher the ratio, the higher the probability of kidney disease due to impaired excretion of creatinine and urea.

The morphological studies of blood at the early lactation period revealed the superiority of Russian Holstein dairy cows supplemented with multicomponent synbiotic “Kormomix® Rumin” in their diets compared to the control group. Thus, we conclude that the inclusion of multicomponent synbiotic “Kormomix® Rumin” in diets of Russian Holstein dairy cows, in general, did not deteriorate the blood characteristics. Our results match those obtained by (27); they mentioned that hematological and biochemical parameters did not vary between dairy cows fed different treatments of *B. subtilis*.

## Conclusion

Inclusion of the multicomponent synbiotic supplement “Kormomix® Rumin” into the ration of the highly productive Russian Holstein lactating cows has no impact on milk productivity in the early phase of lactation (120 DIM). At the same time, it improves the intensity of the rumen fermentation process. Moreover, it does not deteriorate the physiological and health status of the supplemented animals. Consequently, we recommend the inclusion of the multicomponent synbiotic feed additive “Kormomix® Rumin” at (25 g/head/day) as the most appropriate dose in the cows' ration for balancing the diets to optimize rumen's physiological processes. Nevertheless, an economic analysis (cost-benefit) is required in order to, determine if the inclusion of the multicomponent synbiotic feed additive “Kormomix® Rumin” in the rations of Russian Holstein dairy cows is effective or not as well as, further investigation of its effect throughout the entire lactation season (305 DIM) is also needed.

## Data Availability Statement

The raw data supporting the conclusions of this article will be made available by the authors, without undue reservation.

## Ethics Statement

The animal study was reviewed and approved by Bioethics Commission of the Institute of Animal science and Biology of the Russian State Agrarian University – Moscow Timiryazev Agricultural Academy (Protocol № 2021-4 from 12.10.2021).

## Author Contributions

VT, NB, SS, AS, MB, IK, MF, OK, and DA: conceptualization, methodology, formal analysis, data curation, writing—original draft preparation, writing—review and editing, visualization, and supervision. All authors contributed to the article and approved the submitted version.

## Conflict of Interest

The authors declare that the research was conducted in the absence of any commercial or financial relationships that could be construed as a potential conflict of interest.

## Publisher's Note

All claims expressed in this article are solely those of the authors and do not necessarily represent those of their affiliated organizations, or those of the publisher, the editors and the reviewers. Any product that may be evaluated in this article, or claim that may be made by its manufacturer, is not guaranteed or endorsed by the publisher.
